# Protective Effects of Recombinant Kunitz-Domain 1 of Human Tissue Factor Pathway Inhibitor-2 Against 2-Chloroethyl Ethyl Sulfide Toxicity In Vitro [Retraction]

**Published:** 2016-08-30

Choi MS, Parikh K, Saxena A, Chilukuri N. *Protective Effects of Recombinant Kunitz-Domain 1 of Human Tissue Factor Pathway Inhibitor-2 Against 2-Chloroethyl Ethyl Sulfide Toxicity In Vitro*. 2007;8:23-35

The sponsors of the study cannot assure the validity of the data so we, Moonsuk S. Choi, Kalpana Parikh, Ashima Saxena, and Nageswararao Chilukuri, are retracting the paper.

